# An evidence of group discussion and problem-solving skills in MBBS freshers: Implications and applications

**DOI:** 10.30476/jamp.2019.81219.0

**Published:** 2021-01

**Authors:** SANTOSH KUMAR, ZAYABALARADJANE ZAYAPRAGASSARAZAN, DHARANIPRAGADA KADAMBARI

**Affiliations:** 1 Department of Medical Education, Jawaharlal Institute of Postgraduate Medical Education and Research (JIPMER), Puducherry-605006, India

**Keywords:** Teaching, Problem solving, Undergraduate medical education, Learning

## Abstract

Teachers need to follow adult learning principles and students need to have discussion and problem-solving skills for the use of active learning methods in classroom. We aimed to find out whether MBBS freshers had group discussion and problem-solving skills. A free group discussion session for problem-solving using adult learning principles was held during the foundation course for MBBS freshers. The task for group discussion was to give suggestions to improve the situation indicated by the given general medical professional problem. Qualitative deductive thematic analysis was used to analyze suggestions which could be grouped under the themes of seven roles of CanMEDS Physician Competency Framework. The results of deductive thematic analysis of suggestions of student groups are described under seven themes of the roles of the CanMEDS Physician Competency Framework. MBBS freshers showed excellent group discussion, plenary presentation, and problem-solving skills for the given problem. They also had relevant general knowledge about medical profession. Their problem-oriented thinking skills were facilitated using adult learning principles. It is recommended that the use of group discussion for problem-solving with facilitation by using adult learning principles should be emphasized in undergraduate medical education to promote the use of active learning methods in classroom.

## Introduction

The use of lectures continues to be the norm for undergraduate medical education in India as in the rest of South Asia ( 1 ). Teachers are used to giving lectures and students are used to listening to lectures. However, active learning methods ( 2 ) including problem-based learning ( 3 , 4 ), case-based learning ( 4 ) and team-based learning ( 5 ) are essential for developing higher cognitive skills. For using active learning methods in classroom, teachers should use adult learning principles ( 6 ) including problem orientation and giving autonomy and respect to students, and students should have discussion and problem-solving skills. Discussion skills include skills of exchanging information in a group for a purpose such as problem-solving, and problem-solving skills include those of understanding the problem and finding solutions for the problem. Problem-solving needs background information about the problem. 

**Assessing the problem-solving skills of MBBS freshers**

To assess discussion and problem-solving skills of MBBS freshers (MBBS is the term used in India for medical graduates and only after completion of MBBS they are called GP), we planned a group discussion session for problem-solving. A general medical professional problem, which needed only general knowledge about medical profession for its solution, was used for group discussion. MBBS freshers were expected to possess such general knowledge. Free group discussion method, in which teachers act only as observers, was used to give full autonomy to students and bring out a true picture of their discussion and problem-solving skills. Teachers also used adult learning principles during the discussion. Free group discussion ( 7 ) session with plenary presentations was held in a lecture theatre for 150 MBBS freshers on the last day of three and a half-week foundation course before the beginning of Phase I MBBS course. To bring the best out of students, we created a respectful and non-threatening atmosphere during the group discussion according to adult learning principles ( 6 ). The teacher-facilitator and the other two teachers (the three authors) acted only as observers to give autonomy to students as per adult learning principles. The general medical professional problem selected for discussion was in the form of a recent brief report of an all-India survey released by the Indian Medical Association (IMA) entitled “Majority of doctors in India fear violence”, says IMA survey in TamilNadu print edition of The Hindu dated 03.07.2017. The students were requested to divide in situ into approximately equal eight groups and each group was given copies of the published report of IMA survey. Each group was requested to discuss the given findings of IMA survey and give “suggestions to improve the situation indicated by the report of IMA survey.” Initially, 30 minutes were given and later further 15 minutes were given to enable all groups to complete the discussion. All groups were requested to write down the suggestions on paper anonymously and present them in the plenary. No other information of participants such as identification or demographic information was elicited or recorded. During the plenary session, each group was appreciated and thanked for their suggestions and presentations and no other comments were offered by the teachers. One additional presentation each from three groups was also accepted. All the papers having written suggestions were collected for analysis after the plenary session.

After going through lists of suggestions several times, it was found that suggestions pertained to various roles that medical doctors need to play for effective professional work. Hence, the CanMEDS Physician Competency Framework ( 8 ), including the roles of medical expert, communicator, professional, collaborator, leader/manager, scholar and health advocate, which is a widely used competency/outcome framework both for undergraduate and postgraduate medical education was selected for doing qualitative deductive thematic analysis of suggestions ( 9 ). All suggestions could be included under the themes of these seven roles. The role of health advocate included suggestions for the educational system, healthcare system, and governing system. Under each theme, the suggestions were analyzed to bring out the underlying ideas. However, some meaningful suggestions were included as quotes.

**Responses from the learners**

All groups immediately started discussion in which all students looked involved. Excellent coordination was seen in all groups showing quick decision about group moderator/leader. All presentations in plenary sessions were made with confidence and clarity which meant good selection of presenters. All groups submitted written suggestions after plenary presentations, implying good identification of recorders.

The deductive thematic analysis of suggestions of the student groups was described based on the seven roles of the CanMEDS Physician Competency Framework ( 8 ) such as the role of medical expert, communicator, professional, collaborator, leader/manager, scholar, and health advocate.

**Lessons learned**

MBBS freshers showed excellent group discussion skills and attitude. They showed quick and effective self-organization in self-selecting moderators/leaders, presenters and recorders. Their plenary presentation skills were also very good. This may be accounted for by growing emphasis on presentations and group discussions in schools in India. Another factor may be that MBBS freshers being users of internet and social media are very good in communication and participation, which are necessary for group discussion and interactive learning.

Problem-solving skills include skills of understanding the problem and finding solutions for the problem. The group task given to MBBS freshers was to discuss the published brief report entitled “Majority of doctors in India fear violence, says IMA survey” and give suggestions to improve the situation indicated by it. Giving suggestions included the steps of understanding the problem and finding solutions for the problem. It is known that good communication between doctors and patients is associated with increased patient satisfaction ( 10 ). Conversely, poor communication can be associated with patient dissatisfaction and related problems of anger, violence and litigation. Students correctly emphasized in their suggestions that communication process should be improved. The suggestion that doctor-patient communication should be patient-centred and not doctor-centred is in line with current recommendations of putting patient first in communication and medical practice. However, patient dissatisfaction can also be associated with shortcomings in other roles of doctors besides the role of communicator. Students rightly thought in a holistic way and gave suggestions which covered all roles of doctors ( 8 ). Under the role of medical expert there were suggestions emphasizing history taking, judicious use of investigations and patient safety. Suggestions coming under the role of professional included the need for service orientation, ethical practice and care of physician heath. There were some relevant suggestions under the roles of collaborator, scholar and leader/manager. Suggestions for improving medical education system, heathcare system and governing system fell under the role of heath advocate. The role of health advocate needs emphasis and students could realize this.

The fact that the suggestions of students could be grouped under the themes of essential general roles of medical doctors depicted by the widely accepted CanMEDS roles ( 8 ) showed that students fully understood the roles of medical doctors needed to fulfill heathcare needs in India. It also indicated that the general roles of doctors are basically the same all over the world.

Problem-solving skills need relevant knowledge base. MBBS freshers had good general knowledge about medical profession. This indicated their relevant self-learning skills and attitude and the use of appropriate information sources for general knowledge. As the suggestions of students were very relevant, it indicated that they had good problem-solving skills of understanding the given problem and finding solutions for it. It can also be said that MBBS freshers were responsive to social problems and had good reasoning ability and positive attitude to patients. If these attributes are nurtured during MBBS course, these students would develop into very good doctors.

Adult learning principles ( 6 ) of problem orientation, giving autonomy and giving respect appeared to bring out the best in students. Full autonomy was given through the use of free group discussion ( 7 ) in which teachers acted only as observers. Requesting students to give their suggestions for an important medical professional problem at the beginning of their career conveyed that they were being treated as adults and colleagues and with respect. Stimulating problem-solving thinking was the purpose of group task given to students.

## Conclusion

It can be concluded that the given batch of MBBS freshers showed excellent group discussion, plenary presentation and problem-solving skills in the context of a general medical professional problem. They also had relevant general knowledge about medical profession reflecting their good self-learning skills and attitude. Elicitation of problem-oriented thinking skills was facilitated using adult learning principles.

As increasing emphasis on presentations and group discussions in Indian school education is widespread and the present MBBS students, being users of the Internet and social media, are good in communication and participation, so, it is very likely that such students will have group discussion, presentation, problem-solving and self-learning skills. Thus, it is recommended that the use of active learning methods including group discussion, presentation, problem-solving and self-learning should be emphasized for undergraduate medical education in India. To facilitate the use of these methods, we need to follow the adult learning principles.
